# Lactate-induced lactylation: from basic research to clinical perspectives

**DOI:** 10.3389/fphar.2025.1586973

**Published:** 2025-06-13

**Authors:** Henghe Shi, Yifei Zou, Sijie Jin, Junduo Wu, Bin Liu

**Affiliations:** Department of Cardiology, The Second Hospital of Jilin University, Changchun, Jilin, China

**Keywords:** lactylation, lactate, metabolic processes, pathophysiological processes, complex diseases

## Abstract

Lactate was initially considered a metabolic waste product of glycolysis under hypoxic conditions until the emergence of the lactate shuttle hypothesis. The lactate shuttle hypothesis describes the role of lactate in the delivery of oxidative and gluconeogenic substrates as well as in cell signaling. Lactate is a key molecule that links cellular metabolism to the regulation of cellular activity. Lactate-induced lactylation was first identified and reported in Nature in 2019 by Zhang et al. Subsequently, many studies on lactylation have been reported. Widely distributed lactylation is involved in a myriad of pathological processes and participates in the development and progression of numerous diseases, offering promising potential for future disease treatments. We comprehensively reviewed and organized the existing literature, detailed the metabolic processes of lactate and lactylation, and summarized the existing research methods on lactylation, aiming to provide direction and convenience for future research in this field. Additionally, we summarized the role of lactylation in various pathophysiological processes and elucidated the relationship between lactate modification and various diseases, as well as the targets and drugs that regulate lactylation, which may enable future clinical interventions.

## Introduction

Lactate is a major end-product of glycolysis and has traditionally been regarded as a metabolic waste product under hypoxic conditions. Further research has shown that lactate can be transported inside and outside the cell by monocarboxylate transporters (MCT) (Felmlee et al., 2020) or transmit signals through the specific G protein-coupled receptor 81 (GPR81) (Brown and Ganapathy, 2020). Increasing evidence suggests that lactate plays an important regulatory role in numerous pathophysiological processes (Chen et al., 2022; Vander Heiden et al., 2009).

Post-translational modifications (PTMs) of proteins refer to post-translational changes that occur at specific sites on proteins, affecting their physicochemical properties, spatial conformation, stability, and interactions with other proteins (Liu Y. et al., 2024). As a core mechanism of epigenetic regulation, PTMs substantially enrich the diversity of protein structures and functions. Common modifications include phosphorylation, methylation, acetylation, glycosylation, and ubiquitination. PTMs are influenced by external environmental factors and metabolic status *in vivo*, and they can also influence the occurrence and development of various diseases (Yao et al., 2024). Therefore, targeting PTMs to influence disease progression has become a prominent research focus in recent years.

In 2019, Zhang et al. first discovered that lactylation of lysine residues in histones derived from lactate is a novel PTM of histones that directly stimulates chromatin gene transcription (Zhang et al., 2019). Further research on lactylation has demonstrated that it is involved in a series of important cellular physiological activities such as metabolic regulation, cell aging, immune response, and autophagy (Liu M. et al., 2024; Dai et al., 2022; Li et al., 2024a; Sun W. et al., 2023). Additionally, the occurrence and development of many diseases, such as tumor proliferation, metabolic disorders, cardiovascular diseases, and nervous system disorders, are also associated with changes in lactylation (Gu et al., 2024; Zhu et al., 2024; Zhou J. et al., 2024; Chen J. et al., 2025). Studies have confirmed that altering the lactylation level of proteins can affect pathological and physiological processes. Therefore, exploring the regulation of lactylation is expected to become a promising therapeutic approach.

This review summarizes the metabolic processes of lactate in the body and provides a comprehensive description of lactylate-induced lactylation, including its occurrence, detection methods, the pathophysiological processes involved, diseases related to lactylation, targets and drugs for regulating lactylation. We aimed to comprehensively and thoroughly elucidate lactylation, providing direction for further research on lactylation and identifying potential targets for disease treatment.

## Lactate

Cells produce energy and biosynthetic materials through various metabolic pathways, and glucose is the main energy source for humans. In the cytoplasm, glucose is converted to pyruvate by a series of glycolytic enzymes. Pyruvate then enters the mitochondria and is converted to acetyl-coenzyme A (acetyl-CoA) by pyruvate dehydrogenase (PDH). Acetyl-CoA enters the tricarboxylic acid (TCA) cycle under aerobic conditions for efficient production. However, under hypoxic conditions such as intense exercise and illness, the body lacks sufficient oxygen to meet the needs of cells leading to the conversion of pyruvate to lactate in the cytoplasm by lactate dehydrogenase (LDH) (Ma et al., 2020). Lactate has two isomers: L-lactate and D-lactate. L-lactate is the major form of lactate in mammals and is commonly found in various cells. Conversely, D-lactate is an atypical metabolite formed from methylglyoxal through the action of glyoxalase and is found at low concentrations in normal cells (Xin et al., 2022). In the body, lactate exists in two different forms depending on the pH of the surrounding environment. Under normal physiological conditions (pH 7.2), lactate exists as sodium lactate. In low-pH environments, such as tumor stroma, lactate exists in its free acid form (Haas et al., 2015). In addition to glycolysis, glutamine catabolism is also a source of lactate (DeBerardinis et al., 2007). Glutamine enters cells through the amino acid transporter ASCT2/SLC1A5 and undergoes a series of biochemical reactions to convert it into glutamic acid, pyruvate, NADPH, and other compounds. Pyruvate is a source of lactate, and through this pathway, glutamine is broken down and metabolized to become a secondary source of lactate in cancer cells.

Traditionally, lactate is considered metabolic waste in low-oxygen environments. Brooks proposed that lactate can be produced under fully aerobic conditions and plays an important role in systemic metabolism, including energy sources, gluconeogenesis, and signaling molecule interactions (Brooks, 2018). For example, when the blood sugar level in the body is insufficient, lactate can fulfill the metabolic needs of the brain (Magistretti and Allaman, 2018). Moreover, it can be converted into glucose through gluconeogenesis to maintain blood glucose levels (Bergman et al., 2000; Emhoff et al., 2013). Lactate is a signaling molecule with autocrine, paracrine, and endocrine-like roles, bridging oxidative and gluconeogenic substrate delivery with cell signaling (Brooks, 2002; Brooks, 2009; Hashimoto et al., 2007). Additionally, lactate is a product of the glycolytic pathway and a substrate for the downstream aerobic pathway (mitochondrial respiration) and can be regarded as the link between glycolysis and aerobic metabolism.

The ability of lactate to cross cell membranes is primarily mediated by MCTs. MCTs belong to the solute carrier 16 gene family; among them, MCT4 mainly promotes lactate efflux, whereas MCT1 and MCT2 mainly promote lactate influx (Pucino et al., 2018; Bergersen, 2015). The synergistic activity of MCT1–4 promotes lactate shuttling between glycolytic and oxidative cells and is a key factor in maintaining lactate homeostasis in various tissues (Rabinowitz and Enerbäck, 2020). MCTs on the cell membrane first binds to free protons, then binds to lactate, and transports it to the other side of the membrane, expelling lactate and releasing protons. MCT restores its initial structure after deprotonation and prepares for subsequent transport (Li et al., 2022a). Lactate transport is influenced by various factors, including lactate concentration, pH gradient, and redox state, which can affect the MCT and lactate shuttle (Harmer et al., 2008). Many diseases, such as congenital hyperinsulinism (Giri et al., 2022), MCT1 deficiency (Schlegel, 2015) and tumor development, are associated with abnormal MCT. In addition, lactate can activate GPR81 to exert signaling molecular effects. GPR81 is highly expressed in muscle tissue, the central nervous system, immune cells, tumor cells, and adipocytes and mediates biological processes such as lactate-induced energy metabolism, lipid metabolism, and inflammation regulation (Brown and Ganapathy, 2020).

**FIGURE 1:**
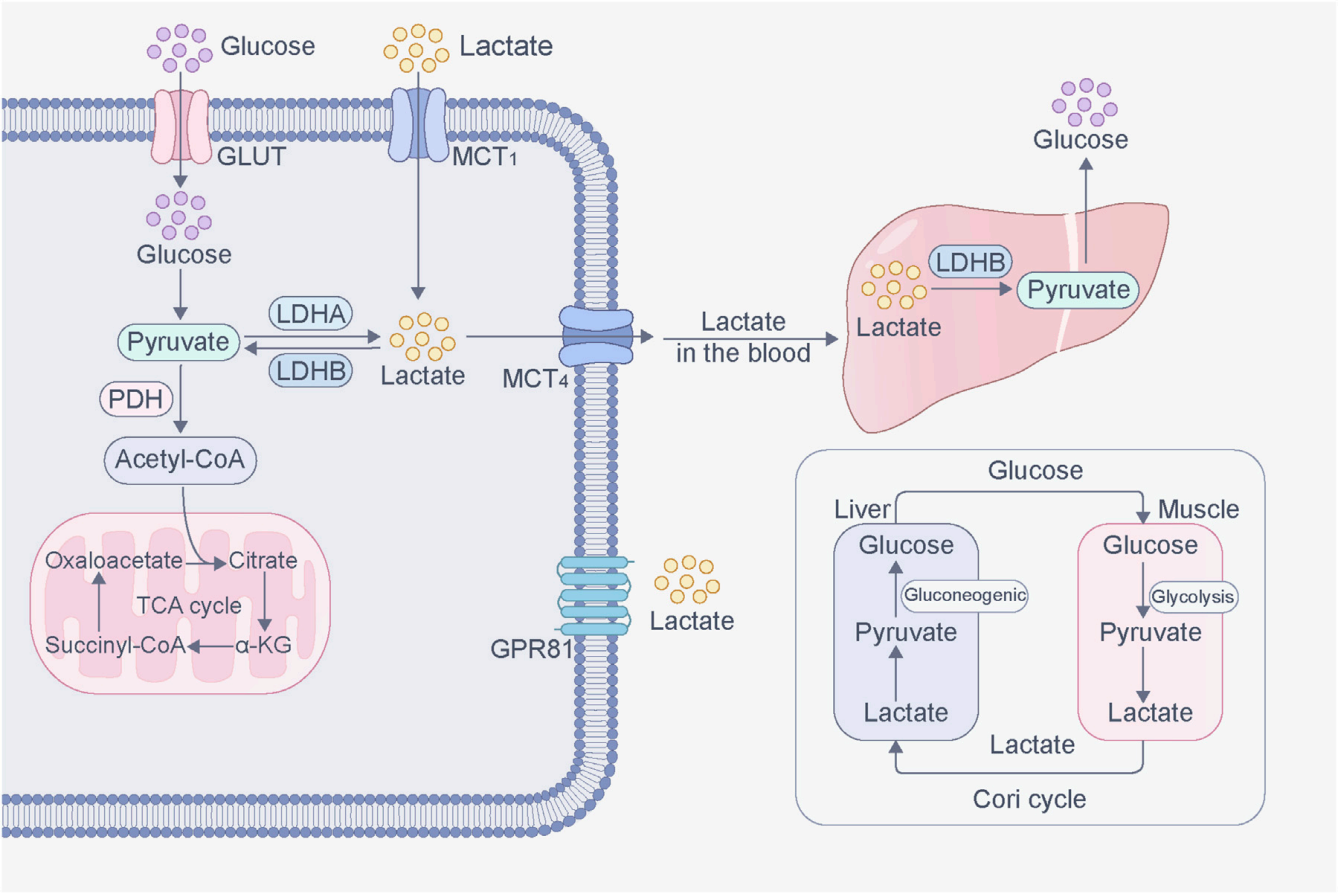
Production and transport of lactate in cells. Glucose is converted into pyruvate through glycolysis. Pyruvate then enters the mitochondria, where it is converted to acetyl-CoA by pyruvate dehydrogenase (PDH), which then enters TCA cycle under aerobic conditions for efficient production. However, under hypoxic conditions, pyruvate is converted to lactate in the cytoplasm by lactate dehydrogenase (LDH). Lactate, inside and outside of cells, can be transported through MCT. In addition, lactate can activate GPR81, initiating signaling molecular effects. Lactate converted from glucose in muscles can be transported to the liver, where it is converted into glucose via gluconeogenesis. This glucose is then released into the bloodstream and reabsorbed by muscles. This process forms a cycle called the lactate cycle (Cori cycle).

Excessive lactate accumulation in the body can lead to lactic acidosis (Hashimoto et al., 2007). Lactate elimination is mainly mediated by two pathways: the TCA cycle and gluconeogenesis. Lactate is converted to pyruvate by lactate dehydrogenase B (LDHB) and then to acetyl-CoA by PDH, which enters the TCA cycle for energy production (Ferguson et al., 2018). Additionally, lactate is the main substrate for gluconeogenesis. In the liver, lactate is converted to pyruvate, and pyruvate is converted to oxaloacetate by pyruvate carboxylase, which undergoes gluconeogenesis to produce glucose (Chandel, 2021). Further, lactate is produced via anaerobic oxidation during muscle contraction. The activity of intramuscular gluconeogenesis is low, so lactate can diffuse into the bloodstream, reach the liver, and produce glucose via the gluconeogenic pathway. Glucose is then released into the bloodstream and reabsorbed by muscles in a process known as the lactate cycle (Cori cycle) (Zhang et al., 2018) (Figure 1).

## Lactylation

In 2019, Zhang et al. first observed and reported lysine lactylation (Kla), demonstrating that the lactylation of lysine residues in histones derived from lactate is a novel epigenetic modification that can directly stimulate chromatin gene transcription and regulate cellular processes (Zhang et al., 2019). The biochemical manifestation of Kla is the addition of 72.021 Da lactyl groups to lysine residues on proteins, which can be detected using high-performance liquid chromatography (HPLC)-tandem mass spectrometry (MS/MS) (Zhang et al., 2019). Moreover, Zhang et al. found that the level of histone lactylation is related to lactate production, and both exogenous and endogenous lactate can lead to histone lactylation formation (Zhang et al., 2019). In addition to histones, increasing evidence suggests that lactylation exists in various non-histone proteins and is a PTMs that occurs when lactyl groups attaches to non-histone proteins. It is commonly found in biological processes such as glycolysis and fat metabolism (Wang J. et al., 2024; Sun et al., 2025). For example, during sepsis, macrophages can absorb extracellular lactate through the MCT to promote high mobility group box-1 (HMGB1) lactylation, thereby increasing endothelial permeability and inducing endothelial barrier dysfunction (Yang K. et al., 2022). In terms of its mechanism of action, histone lactylation regulates gene transcription by regulating the interaction between histones and DNA, whereas non-histone lactylation mainly modifies proteins directly through functional groups, thereby regulating the biological activity and function of proteins. Both are involved in various biological processes.

Since the discovery of lactylation in 2019, numerous researchers have conducted extensive experiments to study its regulatory mechanisms and functions. Similar to other PTMs, the regulation of lactylation is mainly catalyzed by two enzymes: “writers” and “erasers” (Hou et al., 2022). Writers refer to enzymes or proteins that facilitate the interaction of lactyl group with the target molecule to catalyze lactylation. p300 is a classical histone acetyltransferase, and Zhang et al. revealed that p300 acts as a histone lactylation writer, catalyzing the addition of the lactoyl group of lacto-CoA to a specific lysine site (Zhang et al., 2019). Subsequent studies confirmed that p300 is also involved in the lactylation of α-myosin heavy chain (α-MHC) K1897 (Zhang N. et al., 2023). Erasers are enzymes or proteins that can erase lactoyl groups such that the target molecule is de-lactylated and restored to its original state. Moreno-Yruela et al. identified delactylase enzymes capable of cleaving Kla *in vitro*, including class I histone deacetylases (HDAC1–3) and sirtuin 1–3 (SIRT1–3) (Moreno-Yruela et al., 2022; Chen C. et al., 2025). Further studies demonstrated that SIRT1 is a delactylase enzyme of α-MHC K1897 (Zhang N. et al., 2023). By contrast, SIRT3 can delactylate H4K16la, and the processes of delactylation can be directly observed using the chemical probe of p-H4K16la-NBD (Fan Z. et al., 2023). Furthermore, Dong et al. found that YiaC and CobB act as writers and erasers of lysine lactylation in *Escherichia coli* (Dong et al., 2022). These studies confirm that lactylation is mediated by the installation and removal of regulatory enzymes rather than by spontaneous chemical reactions, and the discovery of regulatory enzymes greatly promotes the exploration of the function of lactylation. In addition, proteins that can recognize and bind to specific epigenetic modifications are commonly referred to as “readers.” Recent research has identified Brg1 (Hu et al., 2024), DPF2 (Zhai et al., 2024), and TRIM33 (Nuñez et al., 2024) as readers capable of recognizing lactylation changes in certain proteins. Upon binding, these lactylation readers affect signaling pathways and trigger biological events. However, research on lactylation “readers” remains scarce, and the identification of these readers continues to pose an open challenge.

**FIGURE 2:**
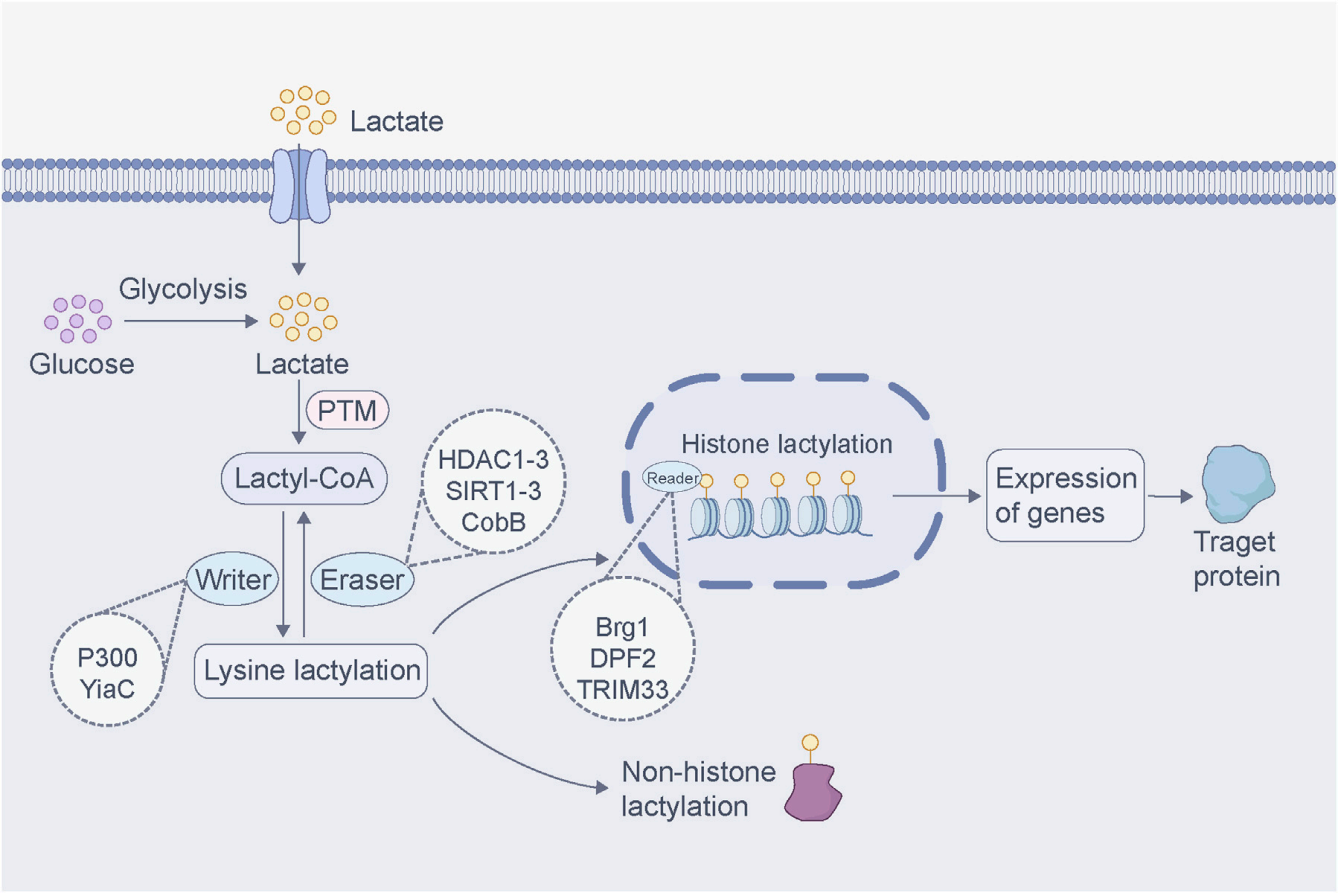
Regulatory mechanisms of lactylation. Lactate produced by glycolysis or entering from outside the cell binds to CoA to form lactoyl-CoA. This lactoyl-CoA acts as a direct substrate for lactylation, where it binds to and dissociates from lysine residues in proteins, a process facilitated by “writer” and “eraser”. The “reader” protein recognizes lactylation changes, influencing downstream signaling pathways and triggering biological events. Histone lactylation regulates gene transcription by modulating the interaction between histones and DNA. In contrast, non-histone lactylation mainly modifies proteins through the addition of the lactoyl group, regulating protein activity and function. Both forms of lactylation are widely involved in various biological processes.

With the advancements in proteomics technology, many lactylation sites have been detected in various species (Li and Zhu, 2020; Lin and Ren, 2024), such as mice (Hagihara et al., 2021), rice (Meng et al., 2021) and *Botrytis cinerea* (Gao et al., 2020). In addition, studies have shown that some proteins undergo lactylation; however, their lactylation sites are currently unknown. For example, Irizarry-Caro et al. found that the Toll-like receptor signaling adapter B-cell adapter for PI3K can regulate the transition of inflammatory macrophages to reparative macrophages by promoting histone lactylation, but the histone modification sites have not been elucidated (Irizarry-Caro et al., 2020). Lactylation is a new PTM that overlaps multiple acetylation modification sites on histones (Gao J. et al., 2023). Moreover, several enzymes that catalyze lactylation are closely related to acetylation, suggesting a complex relationship between lactylation and acetylation (Ren et al., 2025). Other PTMs, such as phosphorylation (Maschari et al., 2022), methylation (Wu D. et al., 2024), and butyrylation (Liu et al., 2022), also interact with lactylation. The functional crosstalk between various PTMs is also an important area for future research. Lactate binds to CoA via high-energy thioester bonds to form lactoyl-CoA. Lactoyl-CoA is a direct substrate for lactylation and forms reversible covalent bonds with lysine residues in proteins. Furthermore, Trujillo et al. found that lactoylglutathione (LGSH) is involved in mediating histone lactylation (Trujillo et al., 2024). LGSH transfers its lactyl group to CoA to form lactoyl-CoA, which facilitates histone lactylation (Trujillo et al., 2024). Both lactate and LGSH can induce lactylation; however, the results may differ. Studies have shown that lactate-induced lactylation promotes a macrophage shift to a repair phenotype (Wang J. et al., 2022), whereas LGSH promotes inflammatory signaling in macrophages via histone lactylation (Trujillo et al., 2024). These results suggested that the effects of lactylation on cells depend on the specific mechanism involved. Future studies will require in-depth structural and functional analyses of lactylated proteins to better understand their structural regulation and biological functions (Figure 2).

## Detection of lactylation

Lactylation has a dose-dependent effect, with elevated intracellular lactate levels inducing lactylation. Lactate concentration can be measured to predict the occurrence of lactylation. Mature test kits are now available for lactate detection. These kits primarily rely on colorimetric methods to determine lactate concentration and can be tested on a variety of samples such as cells, tissues, and blood. For example, a kit test using a colorimetric method showed that the serum lactate level of patients with sepsis-associated lung injury was notably higher than that of healthy controls, facilitating a study on the relationship between lactylation and sepsis-associated lung injury (Wu D. et al., 2024).

Antibodies for detecting lactylation are well established; there are antibodies for detecting all lactylations, such as anti-Pan Kla, and site-specific lactylation, such as anti-H3K14la and H3K18la. Depending on the experimental requirements, Western blot (WB), immunofluorescence, and immunohistochemistry can be used to examine lactylation. They are simple to operate and have low sample preparation requirements. For example, WB showed markedly elevated levels of Pan Kla in the kidneys of a mouse model of diabetic kidney disease (DKD), with a major band at approximately 17 kDa, which may represent H3 (Zha et al., 2024). Histone lactylation changes in DKD were subsequently determined via WB detection of lactylation levels at different residues (H3K18la, H3K14la, H3K9la, H3K23la, H3K27la, and H3K14ac) (Zha et al., 2024).

Mass spectrometry (MS) is a method used to detect moving ions by separating them according to their mass-to-charge ratios using electric and magnetic fields. The exact mass of the ions is measured to determine their compound compositions. MS is the method of choice for lactylation detection because of its objectivity, comprehensiveness, and accuracy (Noberini and Bonaldi, 2023). MS can detect any type of histone PTM in a single experiment without prior knowledge of the modification type or location and reduces antibody-associated cross-reactivity and epitope masking (Karch et al., 2016). Liquid chromatography-tandem mass spectrometry (LC-MS/MS) is an MS-based method that is widely used in large-scale quantitative analysis of proteins and their PTMs owing to its high selectivity and sensitivity (Geffen et al., 2023). For example, histone lactylation was originally discovered when Zhang et al. observed a 72.021 Da mass change in lysine residues in three protein-hydrolyzed peptides, which were identified via HPLC-MS/MS of tryptically digested core histones obtained from MCF-7 cells (Zhang et al., 2019). In 2022, Wan et al. found that cyclic immonium ion of lactyllysine appeared in the MS/MS, enabling reliable lactylation detection and the identification of new lactylation sites (Wan et al., 2022). Additionally, fluorescent probe labeling has also been used to study lactylation. Using this method, Fan et al. developed a p-H4K16la-NBD probe that directly detected the process of erasing Kla through fluorescence (Fan Z. et al., 2023). However, the specificity and sensitivity of these molecules and fluorescent probes for large-scale detection and identification require further investigation.

In addition to traditional methods, applying artificial intelligence (AI) to analyze protein sequences and predict lactylation can quickly and effectively identify potential lactylation sites for further experimentation. The currently developed tools for predicting lactylation include FSL Kla (Jiang P. et al., 2021), Deep Kla (Lv et al., 2022), Auto Kla (Lai and Gao, 2023), ABFF Kla, and EBFF Kla (Yang Y. H. et al., 2024). The development and application of these tools have substantially promoted research on lactylation, such as Deep Kla for lactylation site prediction in rice, by combining a supervised embedding layer, convolutional neural network, bidirectional gated recurrent units, and an attention mechanism layer (Lv et al., 2022). Auto Kla predicts the lactylation site in gastric cancer cells using automated machine learning (Lai and Gao, 2023).

With technological advancements, an increasing number of lactylation detection methods are expected to emerge. Large-scale detection and identification of lactylation rely largely on AI. AI is expected to play an increasingly important role in lactylation analysis by providing technical support for large-scale lactylation research.

## Lactylation and pathophysiological processes

### Metabolism

Lactate produced by glycolysis induces protein lactylation, and most enzymes involved in glycolysis can be modified by lactylation, forming a feedback loop (Dai et al., 2022). The K147 site of the glycolytic enzyme fructose diphosphate aldolase A and the K343 site of α-enolase undergo lactylation and inhibit glycolysis to reduce lactate production (Wan et al., 2022). Lactate inhibited glycolysis and promoted the TCA cycle in non-small cell lung cancer cells (Jiang J. et al., 2021). The mRNA levels of glycolytic enzymes (hexokinase 1 [HK1] and pyruvate kinase M [PKM]) and TCA cycle enzymes (succinate dehydrogenase complex flavoprotein subunit A [SDHA] and isocitrate dehydrogenase [NAD (+)] 3 non-catalytic subunit gamma gene [IDH3G]) can be downregulated or upregulated by lactate, and increased histone lactylation was observed in promoters of HK-1 and IDH3G via chromatin immunoprecipitation assay (Jiang J. et al., 2021). Contrary to the negative feedback loops described above, Pan et al. showed a glycolysis/H4K12la/PKM2 positive feedback loop in Alzheimer’s disease (AD) (Pan R. Y. et al., 2022). The levels of H4K12la were elevated in microglia, and the elevated H4K12la was enriched in glycolytic gene promoters, which activated transcription to enhance glycolytic activity (Pan R. Y. et al., 2022). These findings suggested that lactate is partly involved in cellular glucose metabolism through the lactylation of histones and non-histones.

Additionally, lactylation plays a regulatory role in fat metabolism. Lactate is a major metabolite of high-intensity interval training (HIIT); Lactylation levels in mice peak 24 h after HIIT and return to a steady state at 72 h (Huang et al., 2023). Chen et al. revealed that lactate produced by HIIT upregulates the lactylation of fatty acid synthase (FASN), inhibiting FASN activity and thereby reducing palmitate and triglyceride levels (Chen et al., 2023). The expression of mitochondrial pyruvate carrier 1 (MPC1) is positively correlated with hepatic lipid deposition in patients with nonalcoholic fatty liver disease, and lipid accumulation is reduced in the livers of MPC1^+/−^ mice fed a high-fat diet (Gao R. et al., 2023). The mechanism involves the knockdown of MPC1, which inhibits FASN activity by upregulating FASN lactylation at K673, resulting in decreased lipid synthesis (Gao R. et al., 2023).

### Cell cycle

Lactate is involved in cell cycle regulation through lactylation. In diabetic retinopathy, H3K18la mediates the upregulation of fat mass and obesity-associated protein (FTO), which promotes cell cycle progression by targeting cyclin-dependent kinase 2 (CDK2) to drive angiogenesis *in vitro*, as well as in mice and zebrafish, leading to diabetic microvascular leakage (Chen X. et al., 2024). Centromere proteins (CENPs) are key mitosis-associated protein complexes involved in filament assembly and chromosome segregation. CENPA is remarkably upregulated in hepatocellular carcinoma and is associated with poor patient prognosis (Liao et al., 2023). The lactylation of CENPA at the K124 site promotes CENPA activation and cooperates with Yin Yang-1 (YY1) to drive cyclin D1 and neuropilin two expressions (Liao et al., 2023), which in turn promotes tumor growth (Liao et al., 2023). Additionally, lactylation sites have been found in some cell cycle-related proteins such as HMGB1, a chromatin-associated nuclear protein critical for regulating cell death and survival, and 14-3-3 proteins, a highly conserved protein family involved in life activities such as mitosis and cell cycle control. Overall, lactylation sites have been identified on HMGB1 and 14-3-3 proteins, and further research is needed to investigate the role of lactylation in the cell cycle regulation by these proteins (Yang K. et al., 2022; Lin et al., 2022a).

### Senescence

Tumor necrosis factor receptor-associated protein 1 (TRAP1) is a metabolic regulator associated with senescence. TRAP1 exacerbates vascular smooth muscle cell (VSMC) senescence by increasing lactylation levels of H4K12 and activating senescence-associated secretory phenotype (SASP) transcription (Li X. et al., 2024). The lactylation of H3K18 in microglia is an important factor in promoting brain tissue senescence (Wei et al., 2023). In addition to the lactylation of histones, the lactylation of non-histones plays an important role in senescence regulation. Studies have shown that glutamine administration in disc degeneration inhibits glycolysis and reduces lactate production, thereby downregulating lactylation of AMPKα, inducing AMPKα phosphorylation, and inhibiting senescence (Zhang et al., 2024). Furthermore, inhibiting lactylation of AMPKα promotes cellular matrix synthesis and autophagy (Zhang et al., 2024). The lactylation of LTBP1 at K752 in fibroblasts induces collagen synthesis to attenuate skin senescence (Zou et al., 2024). Regulation of lactylation may be an effective target for modifying organ senescence.

### Inflammation and immune response

Acidosis caused by lactate accumulation in the extracellular environment is a marker of inflammatory diseases (Chen et al., 2022). Lactate concentration in blood and tissues in the physiological environment is approximately 1.5–3 mM, whereas its concentration in inflamed tissues could range 10–40 mM (Pucino et al., 2019). During sepsis, high levels of lactate induce lactylation of HMGB1 in macrophages, stimulating the exosomal release of HMGB1, which increases endothelial permeability and induces endothelial dysfunction (Yang K. et al., 2022). The lactylation of histone H3K18 is upregulated and induces NF-κB activation via the RhoA/ROCK/ezrin signaling pathway, mediating downstream inflammation and apoptosis (Qiao et al., 2024). Lactate promotes the lactylation of ezrin at K263, whereas the K263 mutation reduces the lactylation of ezrin and attenuates inflammation-mediated kidney injury (Qiao et al., 2024). Other studies revealed that lactylation of histones transforms the macrophage phenotype from an M1 phenotype with high glycolytic/low TCA activity to an M2 phenotype with low glycolytic/high TCA activity. M2-like macrophages secrete large amounts of IL-10 and transforming growth factor-β (TGF-β), which inhibits the inflammatory responses, promoting wound healing and inflammation resolution (Noe et al., 2021; Munder et al., 1999). Lactate promotes the transition of macrophages to a reparative phenotype by regulating the lactylation of PKM2 (Wang J. et al., 2022). Increased forkhead box P3 (Foxp3) expression has been observed in natural killer T (NKT) cells exposed to a high-lactate environment. The lactylation of H3K18 near the transcriptional start site of *Foxp3* may direct the phenotypic transition of NKT-like cells to regulatory T cells (Tregs) (Wang Z. H. et al., 2023). In Tregs, lactate facilitates the lactylation of MOESIN at K72 to regulate the development of Tregs, amplifying Treg-mediated immunosuppression in the tumor microenvironment (Gu et al., 2022).

Lactylation plays an important role in inflammatory diseases by amplifying the inflammatory responses or inducing inflammation. The regulatory effect of Kla on immunity is known as the “lactate clock,” which links metabolism and gene regulation based on temporal patterns (Li et al., 2022a). Lactate-based therapies have been proposed to improve the prognosis of patients with inflammatory diseases. Lactate administration reduced acute pancreatitis severity in a mouse model by inhibiting inflammatory modalities (Li H. et al., 2022). However, the time-dependent mechanism of the “lactate clock” requires further exploration to clarify the effect of lactylation levels on inflammation at different times.

### Fibrosis

Lactate acts as a signaling molecule that enhances pro-fibrotic activity in macrophages via lactylation. A remarkable increase in lactate levels was found in TGF-β1-induced lung myofibroblasts *in vitro* and in bronchoalveolar lavage fluid from mice with pulmonary fibrosis. The increased lactate promotes lactylation of histone via p300, enhances the expression of pro-fibrotic genes in macrophages, and promotes pulmonary fibrosis (Cui et al., 2021). Additionally, particulate matter 2.5 (PM2.5) stimulates macrophages to increase glycolytic activity, and upregulated lactylation of histone increases the secretion of pro-fibrotic cytokines such as TGF-β and vascular endothelial growth factor A by activating pro-fibrotic genes in macrophages. This triggers the epithelial–mesenchymal transition (EMT) in lung epithelial cells, leading to the development of pulmonary fibrosis. Lactate dehydrogenase A (LDHA) inhibitor GNE-140 has been shown to reverse this process (Li J. et al., 2024). Similarly, in the heart and kidney, lactate promotes EMT through the regulation of lactylation, which facilitates fibrosis development (Zha et al., 2024; Fan M. et al., 2023).

Activation of hepatic stellate cells (HSCs) is a major contributor to liver fibrosis following injury. Activated HSCs exhibit high glycolytic activity and lactate production rates; evidence suggests that inhibiting glycolysis prevents HSCs activation (Rho et al., 2023). H3K18la is upregulated in activated HSCs, and either HSC-specific or systemic deletion of hexokinase 2 (HK2) inhibits hepatic fibrosis *in vivo* by reducing H3K18la expression, which is reversed by exogenously supplementing lactate (Rho et al., 2023). Additionally, Wu et al. found that lactylation of H3K18 accelerated liver fibrosis progression by promoting SOX9 transcription (Wu S. et al., 2024). Bioinformatic analysis showed that lactylation is extensively involved in the pathological process of liver fibrosis, highlighting its potential as a new therapeutic target for liver fibrosis; however, more regulatory mechanisms need to be further studied (Li L. N. et al., 2024).

### Autophagy

Autophagy involves the wrapping of cellular contents by lipid bilayer membranes to form autophagic vesicles, which fuse with lysosomes to form autophagic lysosomes and degrade their contents to meet the metabolic needs of the cell and organelle renewal (Dikic and Elazar, 2018). Autophagy of physiological processes is a regulatory factor that maintains tissue homeostasis and function. Excessive autophagy exacerbates tissue damage to form pathological processes.

Glycolysis and autophagy are both highly conserved processes. Sun et al. demonstrated that lactate is the signaling molecule that links the two processes (Sun W. et al., 2023). Their study revealed that strenuous exercise-induced muscle degeneration and mitochondrial damage can be eliminated by autophagy to maintain muscle cell homeostasis. Strenuous muscle exercise generates large amounts of lactate, upregulates lactylation of vacuolar protein sorting 34 (VPS34), increases VPS34 lipid kinase activity, and induces muscle tissue autophagy (Sun W. et al., 2023; Jia et al., 2023). Transcription factor EB (TFEB) is a central regulator of autophagy and lysosomal gene expression; recent studies have shown that lactate molecules can modify TFEB to lactylation, increasing TFEB activity and autophagic flux (Huang et al., 2024). Similarly, enhanced lactylation levels of TFEB have been observed in human pancreatic cancer samples. The lactylation of TFEB may be associated with high levels of autophagy in rapidly proliferating cells (e.g., cancer cells), which requires further exploration (Huang et al., 2024). Additionally, interactions between lactylation and autophagy are involved in tumor drug resistance. Patients with metastatic colorectal cancer exhibit elevated levels of histone lactylation, which promotes the transcription of RUBCNL/Pacer (a rubicon-like autophagy enhancer), facilitating the maturation of autophagosomes through interaction with Beclin 1. Inhibition of histone lactylation and autophagy enhances the sensitivity of metastatic colorectal cancer to bevacizumab treatment (Li W. et al., 2024).

### Pyroptosis

Pyroptosis is a proinflammatory form of regulated cell death. The main features of pyroptosis are the rapid formation of holes in the plasma membrane, cell-permeable swelling, and subsequent lysis, followed by the release of large quantities of cellular components and proinflammatory mediators (Wang et al., 2020). Pyroptosis in the physiological environment plays a key role in host defense against pathogenic infections; however, excessive pyroptosis can result in an inappropriate and sustained inflammatory response, leading to the development of inflammatory diseases. The induction of cellular pyroptosis is a novel anti-tumor strategy.

Lactate is a metabolic by-product of the host and gut microbiota. Increased lactate production using high yields of engineered lactate-producing *Saccharomyces cerevisiae* reduces macrophage pyroptosis and inhibits the release of inflammatory factors to alleviate ulcerative colitis. Further studies on the relationship between lactylation and pyroptosis have been conducted in numerous diseases. Xu et al. found that upregulation of the sex-determining region Y-related HMG-box gene 10 (*Sox10*) is associated with macrophage-like VSMC accumulation and cellular pyroptosis in endothelial hyperplasia *in vitro* and *in vivo* in mice (Xu et al., 2023). The main mechanism is that tumor necrosis factor alpha (TNF-α) drives the transcriptional program of VSMC transdifferentiation through the promotion of lactylation of *Sox10*, leading to cellular pyroptosis (Xu et al., 2023). In drug-induced liver injury, lactate inhibits Caspase-11 ubiquitination by reducing its binding to NEDD4 (a negative regulator of Caspase-11). Additionally, lactate inhibits protein interactions between Caspase-11 and NEDD4 by regulating the lactylation of NEDD4, which accelerates macrophage pyroptosis and exacerbates liver injury (Li Q. et al., 2024). The corticosteroid dexamethasone has been used as a first-line anti-inflammatory agent because of its strong inhibitory effect on inflammation. Recent studies revealed that dexamethasone inhibits pyroptosis by modulating the Hif-1α-glycolysis-lactate axis and protein lactylation, thereby attenuating eosinophilic asthma in mice; this may provide new treatment options for eosinophilic asthma (Chen N. et al., 2024).

### Ferroptosis

Ferroptosis is a newly discovered programmed cell death pathway. It is mainly characterized by an imbalance in intracellular iron metabolism and lipid peroxides accumulation, accompanied by swelling of the mitochondria and rupture of the outer membrane, with subsequent cell death (Dixon et al., 2012; Hassannia et al., 2019). Metabolic processes change dynamically and play key roles in the progression of ferroptosis (Zhang H. et al., 2023).

Elevated lactate levels are important markers of sepsis and are positively correlated with mortality (Nolt et al., 2018). The upregulation of H3K18la in septic mice induces ferroptosis via the methyltransferase-like 3/long chain acyl CoA synthetase 4 (METTL3/ACSL4) axis (Wu D. et al., 2024). H3K18la binds to the METTL3 promoter to regulate N6-methyladenosine (m6A). This m6A modification mediated by METTL3 is enriched in ACSL4 (Wu D. et al., 2024). The application of STM2457, a METTL3-targeted inhibitor, suppresses ferroptosis and attenuates septic lung injury in mice (Wu D. et al., 2024). Tau mutations in mice prevent AD by decreasing the lactylation of tauK677 and inhibiting ferritin autophagy and ferroptosis via the mitogen-activated protein kinase (MAPK) signaling pathway (An et al., 2024). She et al. found that postoperative CK-MB and cTnT levels were notably lower in patients with heart valve disease treated with dexmedetomidine (She et al., 2024). This process involves the downregulation of lactylation of malate dehydrogenase two by dexmedetomidine through the modulation of metabolic reprogramming, inhibiting ferroptosis to improve mitochondrial function and reduce ischemia-reperfusion injury (She et al., 2024).

Lactylation is widely involved in various biological processes (Table 1), the regulatory role of lactylation *in vivo* remains elusive. Unraveling the intricacies of lactylation involvement *in vivo* necessitates meticulous biochemical and genetic investigations.

**TABLE 1:** Relationship between lactylation and pathophysiological processes.

Classification	Disease	Animal	Cell	Lactylation site	Mechanism and outcome	Ref.
Metabolism	NR	NO	Multiple types of cells, such as BV2, RAW264.7	ALDOA K147 ENO1 K343	Reduce the activity of ALDOA and ENO1 Inhibition of glycolysis process	Wan et al. (2022)
	Non-small cell lung cancer	NO	NSCLC	Histone	Inhibit glycolysis while promoting TCA cycle	Jiang et al. (2021b)
	AD	Mice, human	BV2	H4K12	Enhance glycolytic activity	Pan et al. (2022a)
	HIIT	Mice	3T3-L1	FASN	Inhibit FASN activity and reduce lipid synthesis	Chen et al. (2023)
	Nonalcoholic fatty liver disease	Mice	AML-12	FASN K673	Inhibit FASN activity and reduce lipid synthesis	Gao et al. (2023b)
Cell cycle	Diabetic retinopathy	Mice, zebrafish	HUVEC	H3K18	Regulate cell cycle, promote cell proliferation and angiogenesis, and lead to diabetic microvascular leakage	Chen et al. (2024a)
	Hepatocellular carcinoma	Human	HCC	CENPA K124	Promote cell proliferation and tumor growth	Liao et al. (2023)
Senescence	Atherosclerosis	Mouse, human	HVSMC	H4K12	Promotes VSMC senescence by activating SASP transcripts	Li et al. (2024b)
	AD	Mice	BV2	H3K18	Upregulation of SASP expression promotes brain senescence and AD	Wei et al. (2023)
	Intervertebral disc degeneration	Rat human	Human nucleus pulposus cell and rat nucleus pulposus cell	AMPKα	Inhibition of lactylation inhibits senescence	Zhang et al. (2024)
	Skin aging	Mice	Human foreskin fibroblasts	LTBP1 K752	Promotes collagen synthesis in fibroblasts for youthful skin	Zou et al. (2024)
Inflammation and immune response	Sepsis	Mice	Macrophage	HMGB1	Inducing endothelial dysfunction	Yang et al. (2022a)
	Inflammation-mediated kidney injury	Mice	RTEC	H3K18 Ezrin K263	Promote inflammation and worsen kidney damage	Qiao et al. (2024)
	NR	Mice	Macrophage	PKM2 K62	Promote the transformation of macrophages toward a reparative phenotype	Wang et al. (2022a)
	malignant pleural effusion	Human	NKT cells	H3K18	Inhibit immune function	Wang et al. (2023a)
	Liver cancer	Mice	Regulatory T cells	MOESIN Lys72	Inhibit immune function	Gu et al. (2022)
Fibrosis	Pulmonary fibrosis	Mice	Lung myofibroblasts	Histone	Enhances the expression of pro-fibrotic genes in macrophages and promotion of pulmonary fibrosis	Cui et al. (2021)
	Pulmonary fibrosis	Mice	RAW264.7	Histone	Entering pulmonary fibrosis	Li et al. (2024c)
	Cardiac fibrosis	Mice	HUVECs	Snail1	Promoting EMT and cardiac fibrosis	Fan et al. (2023b)
	Renal fibrosis	Mice	HK2	H3K14	Promoting EMT and renal fibrosis	Zha et al. (2024)
	Liver fibrosis	Mice	Primary HSC	H3K18	Promoting Fibrosis	Rho et al. (2023)
	Liver fibrosis	Rat	LX-2	H3K18	Promoting Fibrosis	Wu et al. (2024b)
Autophagy	NR	Mice	Multiple types of cells	VPS34 K356 VPS34 K781	Promoting autophagy	Jia et al. (2023)
	Cancer	Human	HEK293, HEK293T, HeLa and PANC1	TFEB K91	Promoting autophagy	Huang et al. (2024)
	Colorectal cancer	Human, mice	The CRC cells	H3K18	Promoting autophagy	Li et al. (2024e)
Pyroptosis	NR	Mice	VSMCs	Sox10	Promoting pyroptosis	Xu et al. (2023)
	Liver injury	Mice	Macrophage	NEDD4 K33	Promoting pyroptosis	Li et al. (2024f)
	Asthma	Mice	THP-1	NR	Inhibit lactylation and subsequently suppress pyroptosis	Chen et al. (2024b)
Ferroptosis	Sepsis-related lung injury	Human, mice	MLE12	H3K18	Promoting ferroptosis exacerbates sepsis-related lung injury	Wu et al. (2024a)
	AD	Mice	BV2	tau K677	Reduced lactylation of Tau protein inhibits ferritin autophagy and ferroptosis, prevents AD	An et al. (2024)
	Myocardial ischemia-reperfusion	Rat, human	H9c2	MDH2 K241	MDH2 lactylation induces ferroptosis, leading to myocardial ischemia-reperfusion injury	She et al. (2024)

NR: not reported, ALDOA: aldolase A, ENO1: enolase 1, TCA: tricarboxylic acid, AD: Alzheimer’s disease, HIIT: high-intensity interval training, FASN: fatty acid synthase, CENPA: centromere protein A, VSMC: vascular smooth muscle cell, SASP: senescence-associated secretory phenotype, LTBP1: latent-transforming growth factor beta-binding protein 1, EMT: epithelial–mesenchymal transition, VPS34: vacuolar protein sorting 34, TFEB: transcription factor EB, Sox10: sex-determining region Y-related HMG-box, gene 10, NEDD4: E3 ligase neural precursor cell expressed developmentally downregulated 4, MDH2: malate Dehydrogenase 2.

## Lactylation in complex diseases

As a novel PTM, lactylation is involved in the regulation of various physiological mechanisms. The lactylation protein is associated with normal cellular functions, whereas aberrant lactylation is closely related to the development of various diseases as shown in Table 2.

**TABLE 2:** Effect of lactylation in various diseases.

Classification	Disease	Animal	Cell	Lactylation site	Mechanism and outcome	Ref.
Cancer	Ocular melanoma	Human	Ocular melanoma cell lines	H3K18	Promote YTHDF2 expression and tumorigenesis	Yu et al. (2021)
	Clear cell carcinoma of the kidney	Mice, human	HK2 Human RCC cell line	H3K18	Activate PDGFR β signaling to promote tumor development	Yang et al. (2022b)
	Colorectal cancer	Mice, human	Colorectal cancer cells	H3K18	Histone lactylation promoted the transcription of RUBCNL/Pacer, facilitating autophagosome maturation Promote resistance of colorectal cancer to bevacizumab treatment	Li et al. (2024e)
	Lung cancer-derived brain metastasis	Mice	PC9-BrM3	H4K12	Activate CCNB1 transcription to promote resistance to pemetrexed in lung cancer-derived brain metastasis	Duan et al. (2023)
	Breast cancer	Human	MCF7 HCC1806	H3K18	Promote breast cancer cell proliferation through c-Myc-SRSF10 axis	Pandkar et al. (2023)
	Bladder cancer	Human, mice	Multiple types of cells, such as 5,637, T24	H3K18	Regulating LCN2 expression to promote tumor growth	Xie et al. (2023)
	Liver cancer	Mice	HCCLM3 Hep3B	H3K9 H3K56	Promote tumor growth	Pan et al. (2022b)
	Liver cancer	Human, mice	Huh7 Hepa1-6	CCNE2 K348	SIRT3 induces delactylation of CCNE2 K348la and promotes tumor growth	Jin et al. (2023)
Metabolic disorders	Diabetic retinopathy	Mice, zebrafish	HUVEC	H3K18	Upregulate FTO, increase the stability of CDK2 mRNA, and aggravate diabetes retinopathy	Chen et al. (2024a)
	Diabetic kidney disease	Mice	HK2	H3K14	Promote KLF5 expression, promote EMT, and aggravate diabetes nephropathy	Zha et al. (2024)
	HIIT	Mice	3T3-L1	FASN	Inhibit FASN activity, reduce palmitate and triglyceride synthesis, and reduce blood lipids	Chen et al. (2023)
	Osteoporosis	Mice	BMECs	H3K18	Promote the differentiation of BMSCs to osteoblasts and alleviate osteoporosis	Wu et al. (2023)
Cardiovascular diseases	Vascular inflammation	Mice	VSMC	Sox10	Driving transdifferentiation of VSMC to macrophage-like cells to promote atherosclerosis	Xu et al. (2023)
	Atherosclerosis	Mice	MAEC	Mecp2 k271	Inhibits atherosclerosis by attenuating ox-LDL-induced inflammation through the MAPK pathway	Wang et al. (2023b)
	MI	Mice	HUVECs	Snail1	Promotes EMT, exacerbates cardiac dysfunction	Fan et al. (2023b)
	MI	Mice	Monocyte	H3K18	Promotes early remote activation of repair genes in monocytes, facilitating the establishment of immune homeostasis and improving cardiac function after MI	Wang et al. (2022b)
	Heart failure	Mice	H9c2	α-MHC K1897	Promote the interaction between α-MHC and Titin, thereby alleviating heart failure	Zhang et al. (2023a)
Nervous system disorders	Cerebral infarction	Rat	PC12	LCP1	Promotes apoptosis and the progression of cerebral infarction	Zhang et al. (2023c)
	Cerebral infarction	Mice	NO	NR	Aggravating brain damage in cerebral infarction	Xiong et al. (2024)
	cerebral ischemia-reperfusion	Rat	N2a	H3K18	Promote HMGB1 expression and induce pyroptosis, exacerbating brain injury in cerebral ischemia-reperfusion	Yao and Li (2023)
	AD	Mice	BV2	H4K12	Exacerbates the dysfunction of microglia in AD, promoting AD development	Pan et al. (2022a)
	AD	Mice	BV2	H3K18	Through the NFκB signaling pathway, upregulates SASP expression and promotes brain senescence and AD	Wei et al. (2023)
	AD	Mice	BV2	tau K677	Influences iron metabolism through the MAPK pathway and promotes AD development	An et al. (2024)
	Neural excitation	Mice	Cortical neurons	H1	Lead to a decrease in social behavior and an increase in anxiety-like behavior	Hagihara et al. (2021)
Other diseases	Psoriasis	Human	HaCaT	H3K18	The downregulation of H3K18lac is a key factor in the decrease of ADIPOQ levels in patients with psoriasis	Zhao et al. (2024)
	Ischemic retinopathy	Mice	HMC3	YY1 K183	Enhances FGF2 transcription, promoting angiogenesis and causing ischemic retinopathy	Wang et al. (2023c)
	Prostate cancer	Human	HUVECs PC-3 DU145	HIF1α	Enhances KIAA1199 transcription and promotes angiogenesis in prostate cancer	Luo et al. (2022)
	Embryonic development	Mice	Oocytes	H3K18 H4K12	Impede spindle assembly and chromosome alignment, ultimately preventing meiosis in mouse oocytes	Lin et al. (2022b)
	Embryonic development	Mice	Oocytes	H3K9 H3K14 H4K8 H4K12	Enhance early embryonic development	Yang et al. (2024b)

YTHDF2: YTH, domain-containing family protein 2, PDGFR β: platelet derived growth factor receptor β, RUBCNL/Pacer: rubicon like autophagy enhancer, CCNB1: cyclin B1, LCN2: lipocalin-2, CCNE2: cyclin E2, FTO: fat mass and obesity-associated protein, CDK2: cyclin-dependent kinase 2, KLF5: Kruppel-like factor 5, EMT: epithelial–mesenchymal transition, HIIT: high-intensity interval training, FASN: fatty acid synthase, BMSCs: bone mesenchymal stem cells, Sox10: sex-determining region Y-related HMG-box, gene 10, VSMC: vascular smooth muscle cell, Mecp2: methyl-CpG-binding protein 2, MAPK: mitogen-activated protein kinase, MI: myocardial infarction, α-MHC: alpha myosin heavy chain, LCP1: lymphocyte cytotoxic protein 1, HMGB1: high mobility group box-1, SASP: senescence-associated secretory phenotype, AD: Alzheimer’s disease, ADIPOQ: adiponectin, YY1: Yin Yang-1, FGF2: fibroblast growth factor 2.

### Cancer

Lactate is a known source of energy for cancer cells. In 1920, Otto Warburg found that tumor cells prefer glycolysis for ATP production over mitochondrial oxidative phosphorylation, even in the presence of oxygen. This phenomenon is known as the Warburg effect (Warburg et al., 1927), and the metabolic shift may be related to the abnormal proliferation of cancer cells (Liberti and Locasale, 2016). Lactate concentrations can be relatively low or very high in different individual tumors or within the same lesion. High lactate concentrations are mostly associated with rapid tumor growth or distant metastases already present early in the disease (Walenta et al., 2004). Lactate in the tumor environment can shuttle between neighboring cancer cells, surrounding stroma, and vascular endothelial cells, promoting tumor-associated inflammation and acting as a signaling molecule to facilitate tumor cell proliferation and angiogenesis in the tumor environment (Wu et al., 2018; Doherty and Cleveland, 2013).

Recent studies have linked lactylation to tumor progression, highlighting that altered lactylation plays an important role in tumor-related progression (Dai et al., 2022; Li et al., 2024g). For example, elevated levels of histone lactylation have been detected in ocular melanoma and are associated with poor prognosis (Yu et al., 2021). In clear cell carcinoma of the kidney, high levels of histone lactylation indicate poor prognosis (Yang J. et al., 2022). Lactylation levels are more abundant in gastric tumors than in paraneoplastic tissues (Yang D. et al., 2022). Additionally, elevated lactylation levels play a role in tumor drug resistance. Bevacizumab is a first-line treatment for metastatic colorectal cancer, and patients with metastatic colorectal cancer resistant to bevacizumab therapy exhibit elevated levels of histone lactylation, and inhibition of histone lactylation enhanced the anti-tumor effects of bevacizumab in the colorectum (Li W. et al., 2024). Lactylation is also involved in pemetrexed resistance in patients with lung cancer-derived brain metastasis (Duan et al., 2023).

Given the role of lactylation in tumor development, extensive therapeutic studies targeting lactylation against tumors are currently being conducted. Deficiency of p300 inhibits glycolytic processes, and inhibition of p300 provides a potential therapeutic target for limiting tumor growth (Huang et al., 2018). Pandkar et al. demonstrated that oncometabolite lactate enhances breast cancer progression by orchestrating histone lactylation-dependent c-Myc expression. Their study results showed that limiting glycolytic enzyme activity limits breast cancer progression by downregulating histone lactylation-dependent c-Myc expression (Pandkar et al., 2023). Xie et al. found that circXRN2, which negatively regulates glycolysis and lactate production, inhibits tumor cell proliferation and migration both *in vivo* and *in vitro*. The main mechanism is that circXRN2 binds to speckle-type POZ (SPOP) degron to prevent LATS1 from SPOP-mediated degradation, activating the Hippo pathway. The circXRN2-Hippo pathway regulatory axis suppresses tumor progression by inhibiting H3K18 lactylation in human bladder cancer (Xie et al., 2023). The triterpene anti-tumor compound, demethylzeylasteral (DML), inhibits angiogenesis and cell proliferation in a variety of cancers. The anti-tumor effect of DML was demonstrated in a tumor xenograft model in nude mice, mediated by the modulation of *in vivo* H3 lactylation (Pan L. et al., 2022). Additionally, SIRT3, which functions as a lactylation eraser, prevents the growth of hepatocellular carcinoma by mediating the delactylation of cyclin E2 (Jin et al., 2023). The role of lactylation in cancer is multifaceted, and further studies are required to explore its potential in cancer treatment and prognosis. The targeted regulation of lactylation is a promising new strategy for cancer therapy.

### Metabolic disorders

Elevated fasting lactate levels have been observed in patients with obesity and type 2 diabetes; studies have shown that elevated lactate levels are associated with insulin resistance (Crawford et al., 2008; Lovejoy et al., 1992; Zheng et al., 2024). Lactate and lactylation are involved in the development of various diabetic complications. Increased lactate levels in diabetic mice are associated with diabetic retinopathy (Chen X. et al., 2024). Histone lactylation upregulates FTO expression, which controls CDK2 mRNA in an m6A-YTH domain-containing family protein 2 (YTHDF2)-dependent manner, promoting angiogenesis and triggering microvascular leakage in diabetes mellitus (Chen X. et al., 2024). High lactate levels can accelerate the progression of diabetic nephropathy by promoting Kruppel-like factor 5 (KLF5) expression through histone lactylation, thereby facilitating EMT. Lowering lactate levels can remarkably delay the EMT process and ameliorate tubular fibrosis in diabetic nephropathy (Zha et al., 2024). Metformin is a classic drug used for treating diabetes. Zhou et al. found that in zebrafish, metformin reduced neutrophil responses to inflammation and injury by decreasing H3K18 lactylation (Zhou R. et al., 2024). This indicates that metformin has other effects in patients with diabetes besides reducing blood sugar levels.

Dyslipidemia is an important cause of atherosclerotic plaque formation, and lipid control is the primary method of plaque stabilization. Huazhuo Tiaozhi granule (HTG) is a herbal medicinal formula widely used in clinical practice for lipid reduction. Lactylation proteomics showed that 198 proteins were lactylated after HTG application, including histones H2B (K6) and H4 (K80), suggesting that the lipid-lowering effect of HTG may be related to lactylation in hepatocytes (Yin et al., 2023). Additionally, Chen et al. showed that HIIT induces lactylation of FASN, which inhibits lipid synthesis and lowers blood lipid levels (Chen et al., 2023).

The absence of the glycolytic regulator, PKM2, in endothelial cells can impair the differentiation of bone mesenchymal stem cells (BMSCs) into osteoblasts, leading to osteoporosis (Wu et al., 2023). The deficiency of PKM2 decreases lactate secretion by endothelial cells, which subsequently reduces the level of histone lactylation in BMSCs. Histone lactylation is crucial for the differentiation of BMSCs into osteoblasts (Wu et al., 2023). Overexpression of PKM2 in endothelial cells, lactate supplementation, and exercise can restore the phenotype of endothelial PKM2 deficient mice, providing a new research direction for osteoporosis treatment (Wu et al., 2023).

Lactylation is widely involved in the regulation of metabolic diseases, and lactylation-dependent lactate is a metabolic product. However, many aspects of the interaction between metabolic and lactylation remain unknown. Further research targeting lactylation could provide new directions for the diagnosis and treatment of metabolic diseases.

### Cardiovascular diseases

Atherosclerosis is one of the most common cardiovascular diseases. VSMC plays an important role in various cardiovascular diseases, such as atherosclerosis and pulmonary hypertension, owing to their highly plastic phenotypes. Studies have shown that VSMC can change from a contractile to a proliferative phenotype in a lactate-rich microenvironment. This phenotypic shift promotes VSMC migration to the intima, leading to intimal hyperplasia and subsequent stenosis (Yang et al., 2017). Xu et al. revealed that Sox10 lactylation drives VSMC transdifferentiation into macrophage-like cells, leading to vascular proliferation and atherosclerosis (Xu et al., 2023). On the other hand, it was found that in atherosclerotic lesions, methyl-CpG-binding protein 2 (MeCP2) K271 lactylation increases plaque stability and inhibits its development (Wang Y. et al., 2023; Chen L. et al., 2024). Wang et al. found that lysine residues in MeCP2 were lactylated at high levels in the aortic tissues of exercise-trained mice and that lactylation of MeCP2 K271 attenuated ox-LDL-induced inflammation by inhibiting the MAPK pathway to inhibit atherosclerosis development (Wang Y. et al., 2023). Furthermore, Chen et al. revealed that exercise inhibited atherosclerosis and played a cardioprotective role by promoting M2 macrophage polarization through MeCP2 K271 lactylation (Chen L. et al., 2024). Increased lactate-mediated lactylation may be one of the mechanisms underlying the protective effects of exercise. In contrast, Xu et al. reported that exercise decreases myocardial lactylation, thereby downregulating the m6A RNA-binding protein, YTHDF2. Inhibition of YTHDF2 promotes exercise-induced physiological cardiac hypertrophy and attenuates ischemia/reperfusion remodeling (Xu et al., 2024). The different trends in lactylation changes between the two studies may be attributed to the exercise regimen and detection site. Specifically, Chen et al. trained mice to perform 8 weeks of exercise on a motorized rodent treadmill and detected lactylation levels in isolated aortic root plaque macrophages in these mice (Chen L. et al., 2024). Xu et al. subjected mice to a 4-week swimming protocol and detected lactylation levels in myocardial tissue (Xu et al., 2024). This discrepancy in the results highlights the complexity of lactylation regulation *in vivo* and the need to holistically consider the regulation of lactylation.

Vascular obstruction can lead to an increase in the local lactate concentration, which in turn induces lactylation and participates in disease progression. Increased lactate levels after myocardial infarction (MI) promote the upregulation of Snail1 lactylation, which in turn promotes EMT, increases cardiac fibrosis, and exacerbates cardiac insufficiency (Fan M. et al., 2023). Contrastingly, Wang et al. showed that after MI, dysregulation of glycolysis and MCT1-mediated lactate transport promotes histone lactylation, which promotes early remote activation of repair genes in monocytes, facilitating the establishment of immune homeostasis and improving cardiac function (Wang N. et al., 2022). Subsequently, Zhang et al. found that lactylation of the α-MHC lysine 1897 site was markedly reduced in mice and patients with heart failure. Knocking out the *α-MHC K1897* in mice results in impaired interaction between α-MHC and Titin, leading to heart failure. However, upregulation of lactate concentration through the administration of sodium lactate or inhibition of key lactate transporters in cardiomyocytes can promote the lactylation of α-MHC K1897 and the interaction between α-MHC and Titin, thereby alleviating heart failure (Zhang N. et al., 2023). These findings suggest that lactylation may play different roles at different stages of MI and heart failure. Future research should focus on the temporal and quantitative dependence of lactylation.

Pulmonary arterial hypertension (PAH) is the result of numerous factors, and studies have shown that dynamic regulation of m6A affects the expression levels of PAH-related genes (Xu S. et al., 2021). Additionally, inflammatory and immune diseases participate in pulmonary vascular remodeling through cytokine secretion and metabolic reprogramming (Xu Z. et al., 2021). Lactate can affect m6A through histone lactylation, thereby altering mRNA transcription and translation and affecting cell growth and metabolism. Furthermore, lactylation may affect the immune microenvironment by regulating the number and function of immune cells, thereby affecting PAH (Zhao et al., 2023).

### Nervous system disorders

Lactate is a product of brain glycolysis and has been shown to play a role in the development of various brain diseases. In cerebral infarction, the lactylation level of lymphocyte cytotoxic protein 1 (LCP1) is notably increased, and the inhibition of glycolysis can reduce the lactylation level of LCP1, ultimately alleviating the progression of cerebral infarction (Zhang W. et al., 2023). Astrocyte-derived lactate exacerbates the brain damage in mice with ischemic stroke by promoting protein lactylation (Xiong et al., 2024). Additionally, LDHA can promote HMGB1 expression and induce pyroptosis by upregulating H3K18la, thus exacerbating brain injury during cerebral ischemia-reperfusion (Yao and Li, 2023).

AD is one of the most common neurodegenerative diseases worldwide. Pan et al. revealed that the glycolysis/H4K12la/PKM2 positive feedback loop exacerbates microglial dysfunction in AD and that interrupting this positive feedback loop may help in AD treatment (Pan R. Y. et al., 2022). Subsequently, H3K18 lactylation in aging microglia was shown to promote AD development through the NFκB signaling pathway (Wei et al., 2023). Recently, An et al. showed that a mutation at the K677 site in tau proteins reduced the lactylation of tau and inhibited ferroptosis by regulating iron metabolism factors such as nuclear receptor coactivator four and ferritin heavy chain 1, thereby preventing AD (An et al., 2024). In addition to participating in the development of AD, studies have shown that histone lactylation-related genes (*ARID5B*, *SESN1*, and *XPA*) have the potential to become biomarkers of AD (Guo et al., 2024). With further research, the regulation of lactylation may become a new target for AD treatment. Moreover, lactylation in brain cells is regulated by neural excitation and social stress, which increases histone H1 lactylation and can lead to a decrease in social behavior and an increase in anxiety-like behavior in stress models (Hagihara et al., 2021).

### Other diseases

In psoriasis research, many studies have confirmed that changes in lactate and LDH levels are key factors in disease progression. Adiponectin (ADIPOQ), overall lactylation, and histone lactylation (H3K18la) levels are remarkably reduced in the skin tissues of patients with psoriasis. Among these, ADIPOQ has the potential to serve as a diagnostic marker for psoriasis. Improving the overall lactylation or H3K18la levels can increase ADIPOQ protein levels. Chromatin immunoprecipitation can be used to determine the combination of the H3K18la and ADIPOQ promoter regions. The downregulation of H3K18la is a key factor in decreasing ADIPOQ levels in patients with psoriasis (Zhao et al., 2024).

Under hypoxic conditions, the transcription factor YY1 in microglia is lactylated at K183, and high lactylation of YY1 enhances fibroblast growth factor 2 transcription, promoting angiogenesis and causing ischemic retinopathy (Wang X. et al., 2023). In prostate cancer, MCT1 mediates an increase in intracellular lactate, which enhances KIAA1199 transcription and promotes angiogenesis in prostate cancer via lactylation of HIF1α (Luo et al., 2022).

Similar lactylation regulation has been observed in developmental studies. Elevated levels of histone acetylation and lactylation in mice impede spindle assembly and chromosome alignment, ultimately preventing meiosis in mouse oocytes (Lin et al., 2022b). Conversely, Yang et al. found that histone lactylation levels change dynamically during early mouse embryonic development, and supplementing the culture medium with sodium lactate can increase histone lactylation levels and enhance early embryonic development (Yang D. et al., 2024). The differences in these results may be related to the different stages of follicular cell maturation, and the specific mechanisms of acetylation regulation at different stages require further research. Additionally, the level of histone lactylation is elevated in the placenta of patients with preeclampsia, and high levels of histone lactylation promote the upregulation of fibrosis-related genes, which may be a new mechanism for placental dysfunction in preeclampsia (Li et al., 2022c).

## Therapeutic targets and drugs for lactylation

Lactylation is involved in various pathophysiological processes, and increasing evidence suggests that it may be a potential therapeutic target for many diseases, particularly cancer. To date, the regulation of lactylation has mainly focused on glycolysis, MCT, acyltransferases, and delactylases (Table 3).

**TABLE 3:** Therapeutic targets and drugs for lactylation.

Class	Therapeutic targets and drugs	Outcome	Ref.
Glycolysis	Oxygen glucose deprivation	Upregulate H3K9 lactylation	He et al. (2024)
	GSK2837808A	Reduce MOESIN lactylation	Gu et al. (2022)
	GNE-140	Reduce histone lactylation	Li et al. (2024c)
MCT	7ACC2	Reduce H3K18 lactylation	Wang et al. (2023a)
Acyltransferase	Andrographolide	Inhibit p300 activity and reduce H3 lactylation	Wang et al. (2024b)
	C646	Inhibit the enzyme activity of p300/CBP and reduce the lactylation of HMGB1	Yang et al. (2022a)
	A-485	Inhibits p300 activity, reduce YY1 lactylation	Wang et al. (2023c)
	Cholera toxin B subunit	Activates p300 activity and promotes α-MHC K1897 lactylation	Zhang et al. (2023a)
Delactylases enzymes	Trichostatin A and apicidin	Inhibit HDAC1–3 enzyme activity and inhibit histone delactylase	Moreno-Yruela et al. (2022)
	AGK2	Inhibition of SIRT2 activity increases METTL16 lactylation	Sun et al. (2023b)
	EX527	Inhibits SIRT1 activity and increases α-MHC K1897 lactylation	Zhang et al. (2023a)
	3-TYP	Inhibits SIRT3 activity and increases Fis1 K20 lactylation	An et al. (2023)
	SRT1720	Catalyze SIRT1 enzyme activity and reduce α-MHC K1897 lactylation	Zhang et al. (2023a)
	Honokiol	Catalyze SIRT3 enzyme activity and reduce CCNE2 K348 lactylation	Jin et al. (2023)
Indirectly regulating lactylation	Evodiamine	Reduce H3K18 lactylation	Yu et al. (2023)
	Demethylzeylasteral	Inhibit histone H3 lactylation	Pan et al. (2022b)
	Hemin	Increase METTL3 acetylation	Zhang et al. (2023d)
	HK2	Increase H3K18 lactylation	Rho et al. (2023)

CBP: CREB-binding protein, HMGB1: high mobility group box-1, YY1: Yin Yang-1, α-MHC: alpha myosin heavy chain, HDAC: histone deacetylases, METTL16: methyltransferase like 16, Fis1: fission 1 protein, CCNE2: cyclin E2, METTL3: methyltransferase like 3.

### Glycolysis

Glucose is converted to pyruvate by a series of glycolytic enzymes, which, in turn, generate lactate through the action of LDH. Lactylation is lactate-dependent, and glycolysis regulates lactate production, thereby regulating lactylation. For example, through oxygen-glucose deprivation, lactate accumulation is induced in microglia, leading to the upregulation of H3K9 lactylation levels (He et al., 2024). Additionally, the glycolysis inhibitors 2-deoxyglucose (hexokinase inhibitor) and dichloroacetate (PDH kinase inhibitor) can lead to a decrease in lactate. Therefore, they are possibly to regulate lactylation (Li et al., 2022a; Zhang et al., 2014). Lactate production depends on the action of LDH, which has four genotypes: LDHA, LDHB, LDHC, and LDHD, which are mainly expressed in skeletal muscles and preferentially convert pyruvate to lactate, whereas LDHB is mainly expressed in the heart and preferentially converts lactate to pyruvate (de la Cruz-López et al., 2019; Valvona et al., 2016). Therefore, LDHA is an important target for the regulation of lactylation. Tumor metabolite lactate promotes tumor growth by regulating MOESIN lactylation in Treg cells, and the LDH inhibitor GSK2837808A combined with anti-programmed cell death-1 (anti-PD-1) has a stronger anti-tumor effect than by anti-PD-1 alone (Gu et al., 2022). The LDHA inhibitor GNE-140 can substantially reduce the upregulation of histone lactylation mediated by PM2.5, thereby alleviating PM2.5-induced pulmonary fibrosis in mice (Li J. et al., 2024). In addition, LDHA activity is associated with malignant proliferation and invasion of tumors and is a potential drug target for tumor treatment (Miao et al., 2013). FX-11 is a selective LDHA inhibitor that exerts anti-tumor effects by inhibiting glycolysis and reducing lactate production (Mohammad et al., 2019; Le et al., 2010). Overall, lactylation is closely related to the development of tumors, and further research is needed to determine whether the anti-tumor effect of LDHA inhibitor is mediated by the regulation of lactylation.

### MCT

MCTs are mainly responsible for transporting lactate into and out of the cell. Drugs that regulate lactate levels mostly target MCTs. The MCT1 small-molecule inhibitor 7ACC2 remarkably reduces the expression of FOXP3 and histone lactylation in NKT-like cells *in vitro* (Wang Z. H. et al., 2023). Additionally, various MCT inhibitors have been found to effectively inhibit tumor growth. For example, syrosingopine, a dual inhibitor of MCT1 and MCT4, has been shown to strongly increase the sensitivity of tumor cells to metformin, making the combination of syrosingopine and metformin a promising anti-tumor therapeutic strategy (Benjamin et al., 2016). The MCT1 inhibitor AZD3965 has been proven effective and safe for the treatment of breast cancer *in vivo* and *in vitro* (Benyahia et al., 2021). The anti-tumor effects of numerous MCT inhibitors involve regulating the lactate concentration in the tumor environment, but whether this is mediated by lactylation requires further exploration.

### Acyltransferase and delactylases enzymes

Lactylation writers and erasers belong to the acyltransferase and delactylase enzyme families. Regulating these two enzyme families can achieve modulation of lactylation. P300, the earliest acyltransferase discovered by Zhang et al., mediates the lactylation of histones H3 and H4 (Zhang et al., 2019). Andrographolide inhibits H3 histone lactylation by interfering with p300, thereby alleviating aortic valve calcification (Wang C. et al., 2024). Additionally, the p300 inhibitors C646 and A-485 have been shown to reduce protein lactylation levels by inhibiting p300 activity (Yang K. et al., 2022; Wang X. et al., 2023). In addition to acyltransferase inhibitors, acyltransferase activators have also been included in studies on lactylation levels. Cholera toxin B subunit is a p300 activator that can promote lactylation of α-MHC K1897 by catalyzing p300 enzyme activity (Zhang N. et al., 2023). Moreover, studies have shown that KAT5/TIP6 can mediate the lactylation of VPS34 (Jia et al., 2023), and general control non-depressible five can mediate the lactylation of lysine (Li et al., 2023), both of which have the potential to targeted regulate lactylation. HDAC1–3 and SIRT1–3 are known delactylases. The HDAC inhibitors trichostatin A and apicidin can targeted regulate protein lactylation through HDAC1–3 (Moreno-Yruela et al., 2022). AGK2 is a SIRT2 inhibitor that notably enhances lactylation of methyltransferase-like protein 16 (METTL16) and induces copper-mediated cell death (Sun L. et al., 2023). The inhibitor EX527 and agonist SRT1720 of SIRT1, as well as the inhibitor 3-TYP and agonist Honokiol of SIRT3, have all been shown to regulate lactylation by modulating the activity of delactylases enzymes (Zhang N. et al., 2023; Jin et al., 2023; An et al., 2023). With the deepening of research on the relationship between lactylation and disease, acyltransferases and delactylases are expected to become important targets for the regulation of lactylation.

### Indirect regulation of lactylation

Evodiamine, derived from Evodiae fructus, inhibits prostate cancer xenograft growth in nude mice. Evodiamine, an intervention drug for regulating lactylation, can inhibit the lactylation of H3K18, thereby reducing angiogenesis (Yu et al., 2023). DML, a triterpene compound extracted from *Tripterygium wilfordii* Hook F, has been shown to inhibit angiogenesis and cell proliferation in various tumors. Recent studies have shown that DML exerts anti-tumor effects by inhibiting H3 lactylation, thereby suppressing the proliferation and migration of liver cancer stem cells (Pan L. et al., 2022). Hemin increases the stability and expression of METTL3 by mediating its lactylation, whereas upregulated METTL3 induces ferroptosis by increasing the level of the transferrin receptor m6A (Zhang L. et al., 2023). Expression of HK2 in activated HSCs is required to induce gene expression via histone lactylation. Targeted inhibition of HK2 reduced H3K18la expression in HSCs and inhibited liver fibrosis (Rho et al., 2023).

### *Clinical* trials *of lactylation*

Research on lactylation has advanced from basic research to clinical trial phases. In preclinical studies, Zhang et al. demonstrated that NBS1 lactylation is required for efficient DNA repair and contributes to hemotherapy resistance (Chen H. et al., 2024). They also reported that stiripentol, a lactate dehydrogenase A inhibitor, can effectively inhibit NBS1 K388 lactylation and mitigate chemotherapy resistance (Chen H. et al., 2024). Notably, stiripentol is a clinically approved drug for epilepsy. Currently, Zhang et al. have initiated A single-arm, prospective, single-center trial investigating stilbestrol in combination with immune-targeted chemotherapy in patients with peritoneal metastatic carcinoma refractory to conventional treatment (Registration No: ChiCTR2400083649). This trial may offer a promising new approach to tumor treatment. In addition, clinical trials involving an MCT1 inhibitor (AZD 3965; Clinical trial: NCT01791595), pyruvate dehydrogenase kinase inhibitor (dichloroacetate; Clinical trial: NCT01029925), and hexokinase inhibitor (2-Deoxy-D-Glucose; Clinical trial: NCT00096707) have demonstrated that these medications induce changes in lactate levels in the body, with mechanisms potentially involving lactylation.

At present, lactylation research is mainly concentrated in the preclinical stage, and its clinical translation faces many challenges. These include the lack of specific detection tools for lactylation, cross-interference from metabolic pathways, and small sample size in clinical trials. The concept of “new use of old drugs” during the process of lactylation conversion offers the potential to significantly shorten the clinical conversion cycle and accelerate the process, presenting a valuable approach to consider.

## Summary and prospects

Lactate, traditionally considered a metabolic waste product, is the final product of glycolysis. Since the 1970s, the understanding of lactate levels has shifted remarkably. Lactate can serve as a metabolic intermediate for the feedback regulation of metabolic processes, as well as a signaling mediator involved in numerous pathophysiological activities, affecting the occurrence and development of numerous diseases. For example, tumor cells tend to produce ATP via glycolysis, which increases the lactate content in the tumor microenvironment. Lactate is a metabolic substrate that is involved in cancer cell proliferation. In addition, lactate has been shown to contribute to the proliferation, invasion, angiogenesis, immune evasion, and other functions of tumor cells (Brown and Ganapathy, 2020). With advances in the study of epigenetic regulation, a novel lactate-induced PTM, lactylation, has been discovered.

Since Zhang et al. first reported lactylation in 2019, lactylation research has expanded exponentially. However, research on lactylation remains in its infancy. Additionally, lactylation may occur on both histones and non-histones. Lactylation regulates protein function in two main ways: (1) lactylated histones directly bind to promoters, promoting or inhibiting the transcription of certain genes, and (2) the lactoyl group directly modifies certain proteins, regulating their biological activity and function. Given its importance in gene expression and metabolic regulation, lactylation has become a new research hotspot in interventional therapies for various diseases.

Protein lactylation has great potential for future clinical applications. By studying the relationship between protein lactylation and the occurrence of specific diseases, new biomarkers can be developed for early detection, diagnosis, and treatment of diseases. Protein lactylation is involved in regulating the course of diseases, such as tumors, cardiovascular diseases, metabolic diseases, and immune diseases. Changes in disease course can be achieved by targeted regulating the lactylation of key proteins. Future research should focus on the development of drugs or small molecules that targeted regulate lactylation. Notably, as a novel PTM, lactylation has a wide range of effects *in vivo* involving various biological activities in multiple organs. However, it is essential to be cautious about the potential side effects that may arise during targeted lactylation regulation. Thus, personalized medicine may be more suitable for the development of lactylation regulation therapies. Additionally, lactylation is associated with the prognosis of various tumors. Exploring the strong association between lactylation and tumor prognosis may provide a reference and basis for the treatment of patients with clinical cancer.

Targeting the production and transportation of lactate and regulating lactylation levels may provide new strategies for disease diagnosis and treatment. Therefore, gaining a deep understanding of the functions and regulatory mechanisms of protein lactylation is essential. Furthermore, future research should prioritize clarifying the precise thresholds for various reactions triggered by lactate levels and developing more targeted approaches for regulating lactylation, ultimately improving clinical outcomes and minimizing risks.

## Glossary

ACSL4: long chain acyl CoA synthetase 4
AD: Alzheimer’s disease
ADIPOQ: adiponectin
AI: artificial intelligence
BMSCs: bone mesenchymal stem cells
CDK2: cyclin-dependent kinase 2
CENPs: centromere proteins
CoA: coenzyme A
DKD: diabetic kidney disease
DML: demethylzeylasteral
EMT: epithelial–mesenchymal transition
FASN: fatty acid synthase
FOXP3: forkhead box P3
FTO: fat mass and obesity-associated protein
GPR81: G protein-coupled receptor 81
HDAC: histone deacetylases
HIIT: high-intensity interval training
HK1: hexokinase 1
HK2: hexokinase 2
HMGB1: high mobility group box-1
HSCs: hepatic stellate cells
HTG: Huazhuo Tiaozhi granule
Kla: lysine lactylation
KLF5: Kruppel-like factor 5
LCP1: lymphocyte cytotoxic protein 1
LDH: lactate dehydrogenase
LDHA: lactate dehydrogenase A
LDHB: lactate dehydrogenase B
LGSH: lactoylglutathione
m6A: N6-methyladenosine
MAPK: mitogen-activated protein kinase
MCT: monocarboxylate transporter
MeCP2: methyl-CpG-binding protein 2
METTL16: methyltransferase like 16
METTL3: methyltransferase like 3
MI: myocardial infarction
MPC1: mitochondrial pyruvate carrier 1
MS: Mass spectrometry
NKT: natural killer T
PAH: Pulmonary arterial hypertension
PD-1: programmed cell death-1
PDH: pyruvate dehydrogenase
PM2.5: particulate matter 2.5
PTM: post-translational modification
SASP: senescence-associated secretory phenotype
SIRT1–3: sirtuin 1–3
Sox10: sex-determining region Y-related HMG-box gene 10
SPOP: speckle-type POZ
TCA: tricarboxylic acid
TFEB: Transcription factor EB
TGF-β: transforming growth factor-β
TNF-α: tumor necrosis factor alpha
TRAP1: Tumor necrosis factor receptor-associated protein 1
VPS34: vacuolar protein sorting 34
VSMC: vascular smooth muscle cell
WB: Western blot
YTHDF2: YTH domain-containijng family protein 2
YY1: Yin Yang-1
α-MHC: alpha myosin heavy chain
